# Development of a Colorimetric Paper Sensor for Hg^2+^ Detection in Water Using Cyanuric Acid-Conjugated Gold Nanoparticles

**DOI:** 10.3390/molecules28186527

**Published:** 2023-09-08

**Authors:** Febrina Amelia Saputri, Eka Ulya Zubaidah, Amaranggani Wikan Puspita Kenanga, Catur Jatmika, Rimadani Pratiwi, Vinayak A. Dhumale

**Affiliations:** 1 Faculty of Pharmacy, Universitas Indonesia, Depok 16424, Indonesia; eka.ulya@ui.ac.id (E.U.Z.); amarangganiwikan@gmail.com (A.W.P.K.); caturjatmika@farmasi.ui.ac.id (C.J.); 2 Department of Pharmaceutical Analysis and Medicinal Chemistry, Faculty of Pharmacy, Universitas Padjadjaran, Jatinangor 45363, Indonesia; rimadani.pratiwi@unpad.ac.id; 3 Department of Applied Science and Humanities, School of Engineering and Sciences, MIT Art, Design and Technology University, Pune 412201, India; vinayak.dhumale@gmail.com

**Keywords:** Hg^2+^, gold nanoparticles, cyanuric acid, colorimetric detection

## Abstract

Hg^2+^ is one of the most dangerous pollutants that can cause damage to organs and the immune system. The common detection methods of Hg^2+^ require sophisticated instrumentation and a long time for analysis. The purpose of this study was to develop a sensor for the detection of Hg^2+^ using filter paper immobilized by gold nanoparticles (AuNPs) conjugated with cyanuric acid (CA). The clear color change from pink to bluish purple is the response of the CA-AuNPs filter paper sensor to exposure to Hg^2+^. Detection can be observed visually with the naked eye and/or with imageJ software; the detection limit is 0.05 µM. The colorimetric response of the sensor was also selective towards Hg^2+^ after testing with different metal ions. In addition, the response from the sensor was also consistent for lake water samples spiked with Hg^2+^. The results of this research provide a promising basic technology for the development of sensors that are affordable, fast, portable, and easy to use for the detection and monitoring of Hg^2+^ levels in water.

## 1. Introduction

Mercury is considered the most toxic heavy metal in the environment [1]. Its exposure can damage the brain, kidney, nervous system, immune system, and endocrine system in humans and animals [2]. Therefore, a sensitive and selective method for detecting the presence of Hg^2+^ in water is needed. Various methods that can be used to detect Hg^2+^ include atomic absorption spectroscopy, fluorescence spectroscopy, UV–Vis spectrophotometry, inductively coupled plasma mass spectrometry, resonance scattering spectroscopy, and electrochemical methods [3,4,5,6]. Although highly sensitive and selective, these methods require highly sophisticated instruments, are expensive, time-consuming, and are not suitable for on-site analysis [7].

Colorimetric methods based on gold nanoparticles (AuNPs) have emerged as a promising field in the last three decades due to their ease of use, low detection limit, and inexpensive cost. AuNPs can be the basis for detecting Hg^2+^ due to their strong surface plasmon resonance (SPR) properties and large absorption coefficient, making them very sensitive to size, shape, surrounding media, and distance between particles [7].

Many colorimetric methods based on AuNPs for Hg^2+^ detection have been developed. Among them, DNA/AuNPs-based colorimetric methods have been used extensively in constructing Hg^2+^ sensors by exploiting the specificity and strong coordination of thymine to Hg^2+.^ As previously reported, Hg^2+^ ions can be detected via the formation of a thymine-Hg^2+^-thymine (T-Hg-T) structure. However, the method using DNA is quite expensive. Functionalization of AuNPs with DNA is also time-consuming and complex. The enzymatic DNA degradation is also unavoidable [8,9]. On the other hand, cyanuric acid (CA) is a compound that has similar structure to thymine, which has a diimide group. The diimide group in CA in the form of heterocyclic N can replace the citrate ion from AuNPs as a stabilizer [9].

Colorimetric paper sensors have garnered a lot of interest in recent years for point-of-care testing and field monitoring because of their low cost, light weight, ease of manipulation (e.g., folding, cutting, and patterning), and minimal sample/reagent consumption [10,11,12,13,14,15,16]. Herein, a paper-based sensor for the easy detection of Hg^2+^ in aqueous samples has been developed. CA can stabilize AuNPs against aggregation, and then immobilize it in a filter paper. When the CA-Hg^2+^-CA complex is formed in the presence of Hg^2+^, the stability of CA-AuNPs decreases, and subsequently AuNPs aggregation occurs. As a result, the color of the paper changes from red to blue, which is easily observed. 

## 2. Results and Discussion

### 2.1. Orientation and Characterization of CA-AuNPs and Detection of Hg^2+^

**Figure 1:**
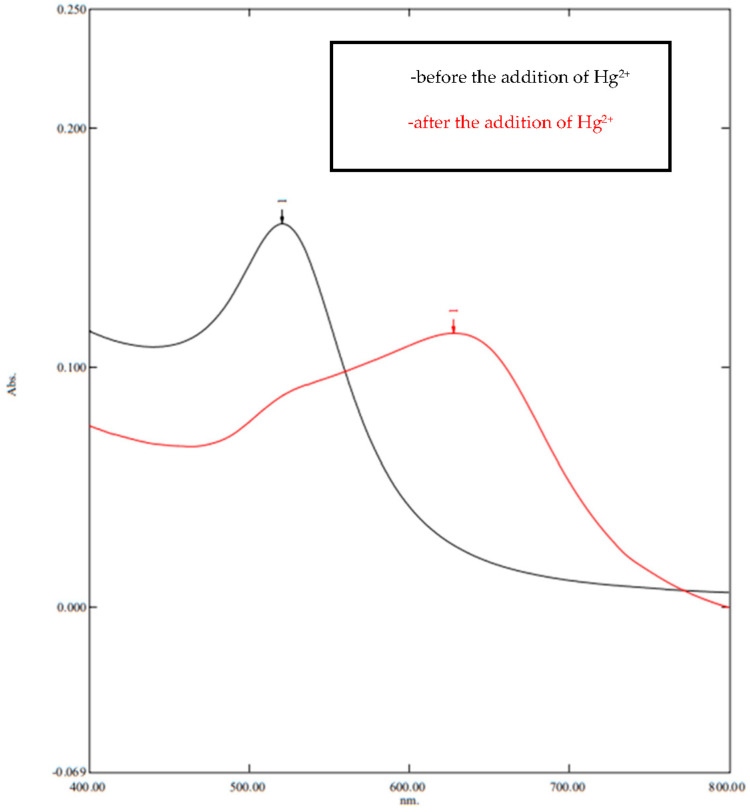
AuNP spectrum before and after the addition of 0.1 M Hg^2+^.

The initial step in this research was to orient the solution of gold nanoparticles conjugated with cyanuric acid to ensure that the solution could be used as a Hg^2+^ metal detector. Gold nanoparticles have different colors according to the particle size. In this study, the AuNPs solution used was obtained from Sigma Aldrich and had a particle size of 10 nm and a red color. The UV–Vis spectrum shows that the gold nanoparticles solution had an absorbance peak at a wavelength of 520 nm (Figure 1).

**Figure 2:**
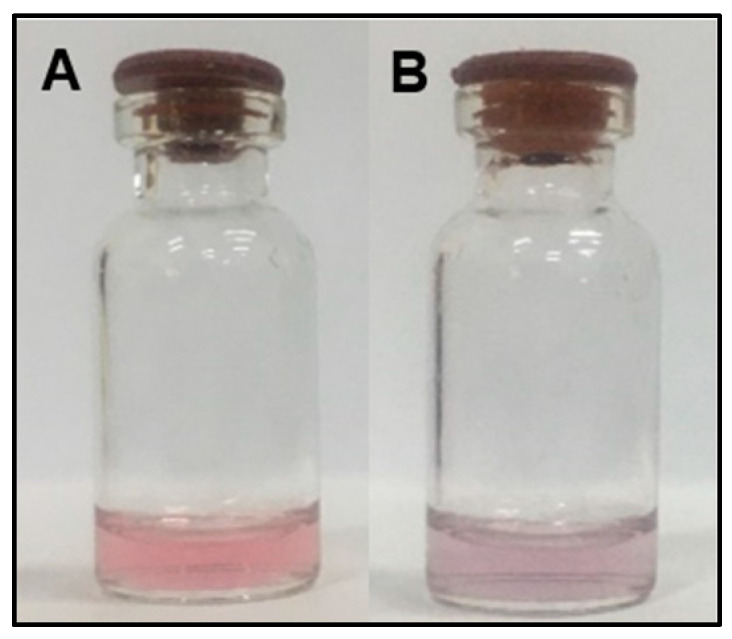
Color of AuNPs solution conjugated with cyanuric acid before (**A**) and after the addition of 0.1 M Hg^2+^ (**B**).

The condition of the acidity level of the solution is important to ensure that the gold nanoparticles solution can react with cyanuric acid and Hg^2+^ [9]. The pH value can affect the detection effect by controlling the morphology and dispersion of the nanoparticles, which can affect the AuNPs SPR band. In addition, changes in pH can change the electrostatic interaction between gold nanoparticles and Hg^2+^. The hydroxyl groups in solution can interact with metal ions or molecules that coat the surface of AuNPs, thereby reducing the coordination effect between metal ions and proteins [17]. This is in accordance with previous studies showing that the diimide group is easily deprotonated under alkaline conditions, thereby affecting the formation of the CA-Hg^2+^-CA structure, the stability of gold nanoparticles, and the results of the colorimetric response [9]. Therefore, 1 mL of 0.1 M Tris HCl buffer with pH 7.4 was used as a buffer solution and added to 600 µL AuNPs solution [9]. Then, 25 µL of 0.1 M cyanuric acid solution was added to the AuNPs solution and incubated for 10 min to ensure the reaction occurred. The resulting solution was a stable pink solution (Figure 2A). Based on visual observations, the color change did not occur significantly when the AuNPs solution was modified. Cyanuric acid has a N group which will bind to AuNPs and become a conjugate on the surface of the gold nanoparticles.

The addition of high salt concentrations can affect the stability of gold nanoparticles due to its charged ion that reacts with gold nanoparticles, thereby mediating CA stabilized-AuNPs aggregation after the addition of Hg^2+^ ions [18]. In this study, 100 µL of 0.5 M NaCl was then added to the solution before adding HgCl_2_ to produce a faster reaction. The addition of NaCl to CA-AuNPs did not directly aggregate the nanoparticles and only slightly disturbed the stability of the AuNPs. The nanoparticles solution that was conjugated with cyanuric acid produces a stable solution even after adding 100 µL of 0.5 M NaCl. This is because cyanuric acid increases the anti-aggregation ability of AuNPs in the presence of heterocyclic N groups [9]. After the addition of HgCl_2_, the solution changed to a bluish-purple color (Figure 2B) and had an absorbance peak at 630 nm on the UV–Vis spectrum (Figure 1). The shift in the absorbance peak and the color change occurred due to the aggregation of CA-AuNPs. This is in accordance with a study conducted by Liu et al. (2012), who has proven the aggregation of CA-stabilized AuNPs solution when Hg^2+^ ions was added [9]. Hg^2+^ has high affinity to the diimide groups from CA, reduces the stability of CA-AuNPs, and initiates Van der Waals forces between nanoparticles which causes aggregation that induces color changes from pink to purple [9,19,20]. This mechanism is also applied and observed in color changes of paper sensor immobilized CA-AuNPs after the addition of Hg^2+^.

### 2.2. Optimization of Colorimetric Paper Sensor

#### 2.2.1. Optimization of Paper Types

Paper selection is primarily based on the fabrication steps required in developing a particular device and application. Chromatography and filter paper are often used. This is related to the high concentration of alpha cellulose, the most stable form of cellulose, which results in a homogenous substrate with a smooth surface. The cleanliness of the paper is high, due to its small or non-existent ash concentration [21]. The selection of the chromatography and filter paper is based on parameters such as porosity, particle retention, and flow rate [22]. The Whatman filter paper types used in this study were Whatman No. 1, Whatman No. 41, Whatman No. 91, and Whatman Chromatography paper No. 1. Each type of paper has a different pore size so that the ability to penetrate liquids varies. The gold nanoparticles used in this study were gold nanoparticles with a size of 10 nm. Therefore, filter paper that has an appropriate pore size will optimally absorb the gold nanoparticle solution and show a clear color. The results of digital imaging from soaking with various papers can be seen in Table 1.

Visually, the Whatman No. 1 had the most vivid color. This is indicated by the results of digital imaging which shows that the Whatman filter paper No. 1 had the smallest greyscale intensity and the greatest absorbance, namely 103.231 and 0.3927 when 0 M Hg^2+^ was added, and 105.800 and 0.3821 when 0.1 M Hg^2+^ was added.

The grayscale intensity value indicates that the Whatman filter paper No. 41 and Whatman Chromatography paper No. 1 has an almost white color and is not clearly visible, while the Whatman filter paper No. 1 and No. 91 shows a small grayscale intensity and large absorbance, which means the paper shows a darker and more visible color. This difference occurs due to differences in the absorption ability of each paper, which depends on the pore size of the paper. The smaller the pore size, the more reagents will be absorbed [23]. If the pore diameter is too large and the specific surface area is small, the absorption capacity will be low. However, if the pore size is too small, the paper will limit the diffusion of the adsorbate and solvent, which increases the shielding effect for molecules with larger diameters [24]. Whatman paper No. 1 and 91 have the smallest pore sizes, namely 11 and 10 µm, so they have the best absorption capacity.

#### 2.2.2. Optimization of CA-AuNPs Paper Soaking Time 

Direct immersion of paper substrates into colloidal nanoparticles has been shown to be effective in manufacturing paper-based devices [25]. Variation of soaking time was carried out in order to obtain the most optimum paper immersion time to be seen visually. Based on the results of visual observations, the pink color on the paper after 3 h and 6 h of immersion was not clearly visible, while it was clearly visible on the paper with an immersion time of 12 h and 24 h. Based on the results of color analysis via ImageJ version 1.54d, the highest absorbance values were found on paper with a 24 h soaking time (Table 2).

In the grayscale image, the intensity of the image is obtained with the white image having the greatest intensity while the black image has the lowest intensity. The results of the paper test with immersion for 24 h showed the lowest greyscale intensity. This shows that the longer the immersion time, the more gold nanoparticles were adsorbed in the paper. Therefore, the selected immersion time was 24 h.

#### 2.2.3. Optimization of CA-AuNPs Paper Drying Temperature

After the paper has been soaked, it needs to be dried before being used as an analytical device. Optimization of drying was carried out at room temperature or 30 °C, and in an oven at 50 °C, 75 °C, and 100 °C. Drying was carried out until the paper was dry, depending on the temperature used. At room temperature, the paper dried in about 1 h, while drying at a higher temperature shortened the drying time. At 50 °C, filter paper dries within 10 min, at 75 °C, the paper dries within 5 min, and at 100 °C, the paper dries within 3 min. Although as the temperature increases the drying time becomes shorter, the stability of CA-AuNPs in paper also needs attention. At temperatures of 75 °C and 100 °C, the analysis device will immediately turn blue due to the instability of AuNPs at high temperatures, so aggregation occurs even though there is no Hg^2+^. Therefore, the optimum drying temperature for CA-AuNPs paper is at 50 °C for 10 min because the nanoparticles are still in a stable form and drying can be done in a shorter time. The observation is based on blue/red intensity, because the final color is purple, which is a mixture of blue and red. Comparison of the intensity of blue and red in paper with and without Hg^2+^ at each drying temperature variation can be seen in Table 3. 

#### 2.2.4. Optimization of Reaction Time

**Figure 3:**
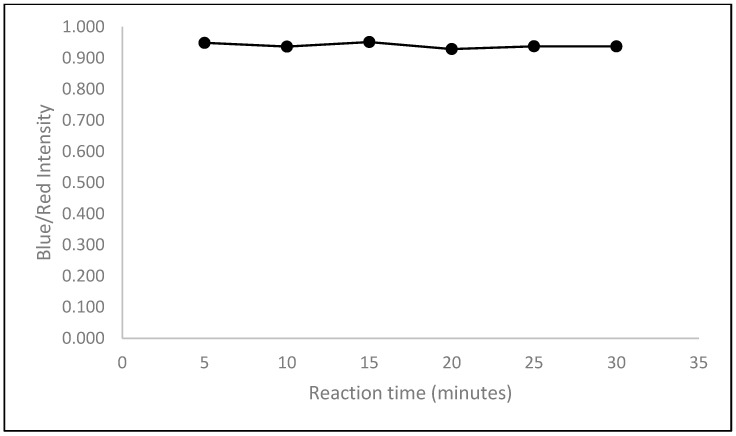
Effect of reaction time on intensity, observed on paper immobilized with CA-AuNPs then added with 0.1 M Hg^2+^.

After soaking the paper in CA-AuNPs solution for 24 h, the paper was dried at 50 °C for 10 min. The dried paper containing CA-AuNPs was then dipped into a 0.1 M Hg^2+^ solution. Then, the reaction time was observed. The color intensity was viewed every 5 min up to 30 min. Visually, the visible color changes with increasing time were relatively very small. Based on ImageJ, the intensity at each time was not much different and did not increase after being left for a longer time (Figure 3). This shows that the gold nanoparticles have been fully aggregated in just 5 min. Therefore, the time chosen for observation was 5 min.

### 2.3. Preparation of Colorimetric Paper Sensor

**Figure 4:**
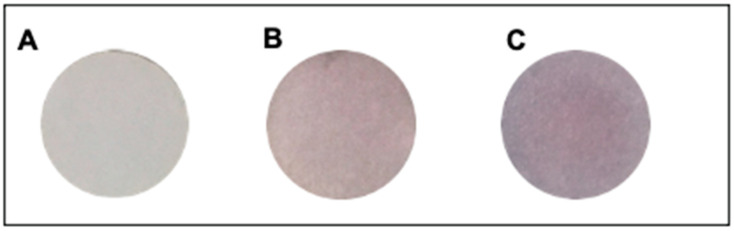
Whatman filter paper color No. 1 before being immobilized by CA-AuNPs (**A**), after being immobilized by CA-AuNPs (**B**), and when the sample was added with 0.1 M Hg^2+^ (**C**).

Based on the optimization results, it can be concluded that the filter paper used should be Whatman No. 1. The paper was cut into circles with a diameter of 1 cm and washed using ethanol, then aquabidest. The washed paper was then dried and placed into the drip plate hole. Each paper was then added with 200 µL of CA-AuNPs solution so that the paper was submerged. Paper immersion in CA-AuNPs solution was carried out for 24 h. After 24 h the CA-AuNPs-immobilized paper was dried in an oven for 10 min at 50 °C. It was proven that the filter paper had been immobilized by CA-AuNPs by the changing of the paper’s color from white to pink (Figure 4).

**Figure 5:**
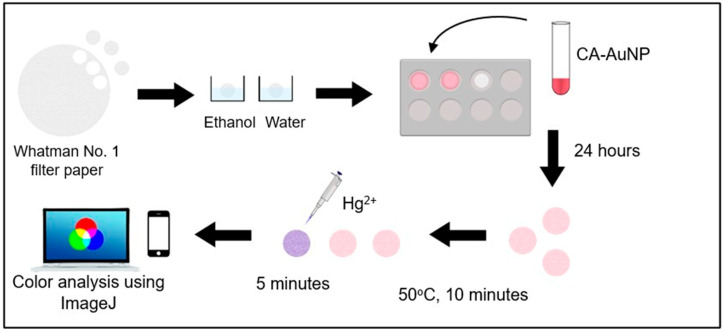
Illustration scheme of paper-based sensor fabrication and testing with CA-conjugated AuNPs.

After the paper was treated, then the analysis test on the water sample can be carried out. The test was carried out by adding a water sample containing 20 µL of Hg^2+^ to the paper set. The color change occurred within the first 5 min. To describe the colorimetric response of the CA-AuNPs-immobilized filter paper to Hg^2+^ in water, the color intensity profile of the resulting digital images was evaluated by measuring the intensity of red, green, and blue (RGB) using ImageJ software. The comparison value of the intensity of blue and red was calculated because it is adjusted to the color of AuNPs before and after experiencing aggregation. A larger comparison value indicates that the blue component is stronger than the red component, which means that AuNPs has undergone aggregation. This characterization approach can be useful in providing semi-quantification of the colorimetric response of the sensor, which can be used for the development of colorimetric sensors in conjunction with spectroscopic techniques [25]. A schematic illustration of the experimental workflow used in this study is presented in Figure 5.

### 2.4. Stability of Colorimetric Paper Sensor

**Figure 6:**
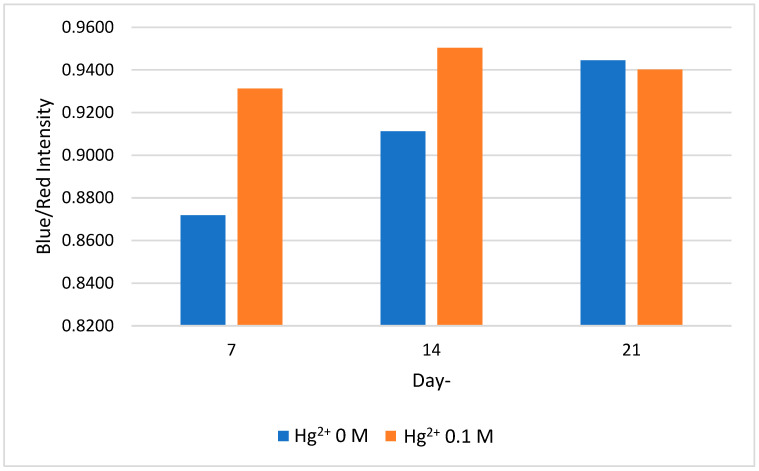
CA-AuNPs paper digital imaging results on days 7, 14, and 21, observed on paper immobilized CA-AuNPs that stored at temperature of 4 °C, then added with 0.1 M Hg^2+^.

Paper device stability was tested by storing the device in a refrigerator with a temperature of 4 °C. Storage was carried out for up to 21 days and test were conducted every 7 days. Based on the results of the study, the CA-AuNPs on the paper remained stable and did not experience aggregation until day 14. Visually, it can be seen that the CA-AuNPs paper set still showed a pink color with the addition of a negative control solution and showed a bluish purple color when added to a positive control solution on the 7th day. On the 14th day, visually, the color was darker but there was still a slight difference in color. However, on day 21, the gold nanoparticles experienced aggregation before the addition of Hg^2+^. The color differences were clarified using ImageJ and a comparison of the intensity of the blue to the red. If seen in Figure 6, the longer the storage time, the higher the ratio of blue to red intensity. This shows that the gold nanoparticles undergo aggregation little by little and experience a shift in SPR.

### 2.5. Selectivity of Colorimetric Paper Sensor

**Figure 7:**
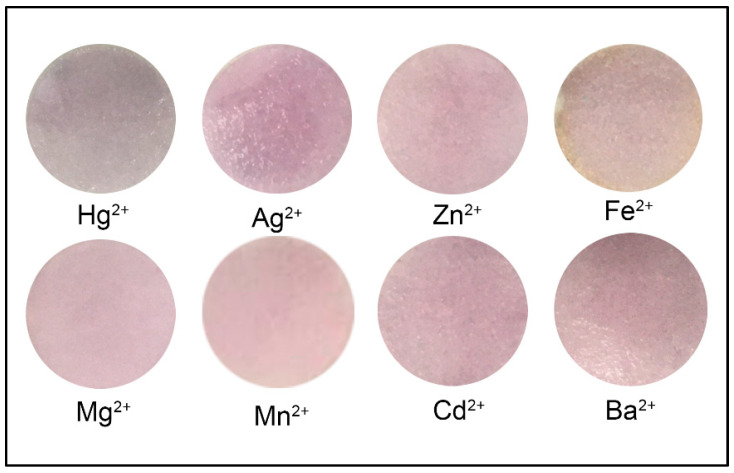
Visual observations of CA-AuNPs-immobilized paper exposed to various metals at a concentration of 0.1 M.

The selectivity of metal ion sensors is very important to explore the application of real sample detection. To realize the selectivity of the system, several common metal ions were selected for investigation, including Ba^2+^, Zn^2+^, Cd^2+^, Mn^2+^, Cu^2+^, Mg^2+^, Ag^2+^, and Fe^2+^ (Figure 7). It was found from the experimental data that each metal ion tested could not induce CA-AuNPs aggregation and no change in absorption was observed within 30 min, even though it was in a large concentration, namely 0.1 M. Meanwhile, the color of the CA-AuNPs paper immediately changed within 5 min only in the presence of Hg^2+^ of the same concentration. The blue/red intensity of CA-AuNPs paper exposed to various metals is shown in Table 4. The highly selective interaction between cyanuric acid and Hg^2+^ results in a decrease in the stability of CA-AuNPs, which is caused by the similarity in structure of cyanuric acid and thymine, which has a heterocyclic N group that will react with Hg^2+^ and can form bridges to connect adjacent cyanuric acid groups to form a CA-Hg^2+^-AuNP structure and result in a shift in the SPR resonance wavelength [26].

### 2.6. Sensitivity of Colorimetric Paper Sensor

**Figure 8:**
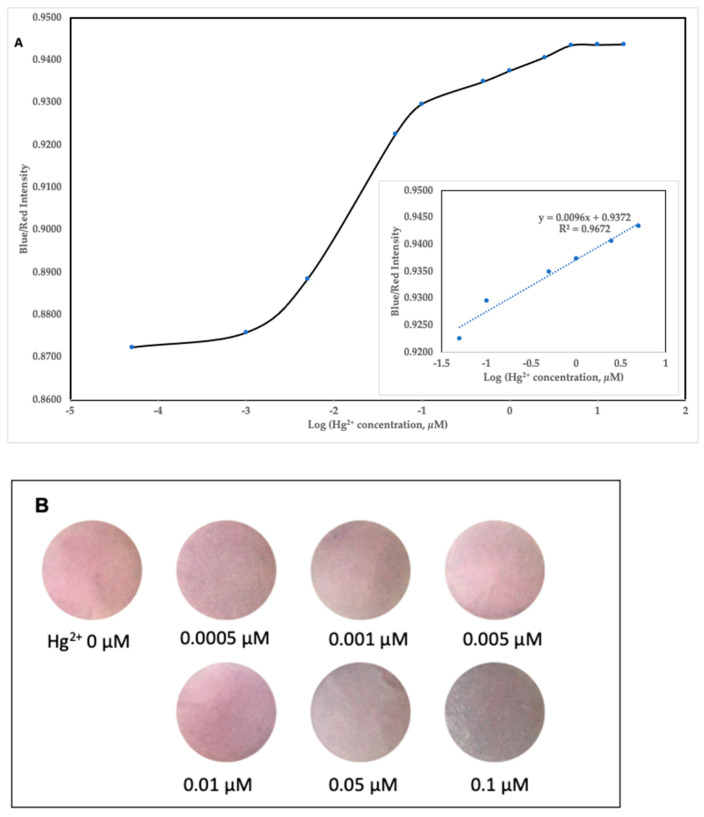
(**A**) Profile of the ratio of the intensity of blue to red at various concentrations of Hg^2+^. (**B**) Visual observation results of CA-AuNP-immobilized paper after exposure to various concentrations of Hg^2+^.

The sensitivity of the colorimetric paper sensor was also evaluated using water with varying concentrations of Hg^2+^ (0.0005–20 µM). Based on the comparison of the intensity of the blue and red colors obtained from ImageJ, there was an increase as the concentration of Hg^2+^ increased (Figure 8A). Based on the graph, the increase continues to occur as the concentration of Hg^2+^ increases, then values gradually reach steady state at a Hg^2+^ concentration of 5 µM. Additionally, the blue/red intensity demonstrates a linear correlation with the logarithm of Hg^2+^ concentration, as can be seen in the inset of Figure 8A. The correlation equation is y = 0.0096x + 0.9372, where “y” is the blue/red intensity and “x” is the Log(Hg^2+^ concentration, µM). The results show that the paper sensor began to show a bluish discoloration at a concentration of 0.05 µM (Figure 8B).

### 2.7. Application of Colorimetric Paper Sensor in Water Samples

To test the applicability of the proposed method, Hg^2+^ in lake water samples was determined under optimum conditions. Water in lakes generally contains various kinds of water contaminants in organic and inorganic forms, which can interfere with the interaction between AuNPs and Hg^2+^ in the water. Therefore, lake water can be used as a test sample to ensure the capabilities of the analytical device being made. The lake water used in this study was lake water from three different lakes located at the Universitas Indonesia, namely Kenanga Lake, FMIPA Lake, and Mahoni Lake. Sample preparation was carried out simply by filtering it through a 0.45 µm membrane before testing. To ensure that the analytical device that has been made can detect Hg^2+^ in a test sample containing other substances, a certain amount of Hg^2+^ was added to the lake water sample. The test results can be seen from Table 5. At a concentration of 0.01 M Hg^2+^ in the three lake water samples, it can be seen that the device was still able to provide an appropriate response, namely a change in aggregation between nanoparticles so that it shows a bluish purple color.

### 2.8. Advantages and Shortcoming of the Method 

Several studies regarding the development of paper sensors based on gold nanoparticles for Hg^2+^ detection have been carried out. Thymine is a common compound used as a stabilizer and detection reagent, because of its selectivity to Hg^2+^ [14,15,16]. However, thymine has weaknesses in terms of stability as well as cost. On the other hand, there is the development of paper sensors without using gold nanoparticles. Hao et al. developed carbon dots using folic acid as a paper sensor for Hg^2+^ with a detection limit of 0.1 µM [27]. In this study, a detection limit of 0.05 µM was obtained. This CA-AuNPs paper sensor has advantages in terms of stability when compared to AuNPs paper sensors with DNA/thymine stabilizers, and in terms of sensitivity when compared to methods without using gold nanoparticles. However, the sensitivity of paper sensors that use DNA/thymine as a stabilizer remains superior, with less stability.

## 3. Materials and Methods

### 3.1. Reagents and Apparatus

UV–Vis spectrophotometry (Thermo Scientific, Waltham, MA, USA), pH meter (Eutech, Breda, The Netherlands), oven (Memmert, Schwabach, Germany) micropipette (DLAB, Beijing, China), beaker (Iwaki, Japan), Erlenmeyer (Iwaki, Japan), test tube (Iwaki, Japan), volumetric flask (Iwaki, Japan), stir bar, vacuum filter, volume pipette (Pyrex, Shizuoka, Japan), analytical balance (Sartorius, Göttingen, Germany), ultrasonic stirrer (Branson, MO, USA), Whatman No. 1, Whatman No. 41, Whatman No. 91, Whatman grade 1 chromatography paper, 0.45 µm membrane, and ImageJ software (National Institutes of Health, Bethesda, MD, USA).

AuNPs (10 nm diameter, OD 1) (Sigma Aldrich, St. Louis, MO, USA), cyanuric acid (Sigma Aldrich), ethanol (Merck, Darmstadt, Germany), double distilled H_2_O (Ikapharmindo Putramas, East Jakarta, Indonesia), HgCl_2_ (Smartlab, Semarang, Indonesia), MgSO_4_, ZnCl_2_, AgNO_3_, FeSO_4_, CdSO_4_, BaCl_2_, and MnCl_2_ (Merck, Germany), NaCl (Merck, Germany), Tris-HCl (Himedia, Kennett Square, PA, USA), as well as water samples from Kenanga Lake, FMIPA Lake, and Mahoni Lake.

### 3.2. Orientation and Characterization of CA-AuNPs and Detection of Hg^2+^

The preparation of gold nanoparticles conjugated with cyanuric acid (CA-AuNPs) was carried out by mixing 600 µL of AuNPs solution with 1 mL of 0.1 M Tris-HCl buffer solution with a pH of 7.4 in a test tube. Then, the solution was incubated with 25 µL of 0.001 M cyanuric acid solution for 10 min at room temperature to obtain a CA-AuNPs solution [21]. CA-AuNPs was then added alternately 100 µL of 0.5 M NaCl and 0.1 M Hg^2+^. The mixture was then shaken until well blended and allowed to react for 20 min at room temperature before the absorbance was measured and the color change observed.

The absorption spectrum was recorded using a UV–Vis spectrophotometer at room temperature. The characterization was carried out twice using CA-AuNPs and CA-AuNPs which had been added with NaCl and 0.1 M Hg^2+^.

### 3.3. Optimization of Colorimetric Paper Sensor

#### 3.3.1. Optimization of Paper Types

The paper used for optimization was Whatman paper with different types and pore sizes. The types of filter paper used were Whatman filter paper No. 1, 42, 91, and Whatman chromatography paper No. 1. The measurement of the color intensity of each paper with a positive control solution of 0.1 M Hg^2+^ was carried out using the ImageJ software version 1.54d.

#### 3.3.2. Optimization of CA-AuNPs Paper Soaking Time 

The immersion time of the filter paper in the CA-AuNPs solution was varied to ensure that the CA-AuNPs adhered to the paper. Immersion was carried out for 3 h, 6 h, 12 h, and 24 h. After soaking, each paper was dried at room temperature and dripped with 20 µL of 0.1 M Hg^2+^, and the color difference was compared with the blank paper. The color differences were distinguished by using the ImageJ software version 1.54d to see the color intensity.

#### 3.3.3. Optimization of CA-AuNPs Paper Drying Temperature

The type of paper that showed the best color was then used for further optimization. Optimization of temperature and drying time was carried out so that paper could be dried efficiently but remain in a stable state. After soaking, the paper was then dried at various temperatures, namely 30 °C, 50 °C, 75 °C, and 100 °C. Drying was carried out within a certain time according to the temperature. The dried paper was then tested using distilled water (blank) and Hg^2+^ solution.

#### 3.3.4. Optimization of Reaction Time

After the analytical device was dripped with 0.1 M Hg^2+^ solution, the reaction time of CA-AuNPs with Hg^2+^ was observed until the color changed. Color changes were observed every 5 min until 30 min.

### 3.4. Preparation of Colorimetric Paper Sensor

The preparation of immobilized CA-AuNPs on filter paper was carried out by cutting the filter paper into circles with a diameter of 1 cm. The circular pieces of paper were then washed with ethanol and water, and then air-dried. The dried filter paper was then placed in the drip plate hole and immersed in 200 µL of CA-AuNPs for sufficient time to ensure immobilization of CA-AuNPs on the filter paper. After soaking, each paper was then dried at the selected temperature for the selected time until dry. The dry paper was ready to be used for sample testing by dripping 20 µL of sample solution.

### 3.5. Stability of Colorimetric Paper Sensor

Selected paper immobilized by CA-AuNPs was then stored in a refrigerator at 4 °C. The analysis was carried out after the paper sets were stored for 7, 14, and 21 days to compare the colors formed after adding only double distilled water and 0.1 M Hg^2+^ solution. The color was then analyzed using ImageJ software to determine the ratio of the intensity of blue to red.

### 3.6. Selectivity of Colorimetric Paper Sensor

To evaluate the selectivity of this system for Hg^2+^, several environmentally relevant metal ions were tested, namely 0.1 M Fe^2+^, Mg^2+^, Zn^2+^, Ba^2+^, Mn^2+^, Cd^2+^, and Ag^2+^. A total of 20 µL of each of these metals was then added to the filter paper modified by CA-AuNPs. After 5 min, the change in color of the paper was observed visually and using ImageJ software.

### 3.7. Sensitivity of Colorimetric Paper Sensor

The sensitivity test was carried out by preparing Hg^2+^ solutions in various concentrations from the range of 0.0005–20 µM. Each concentration of Hg^2+^ was then added by as much as 20 µL to the filter paper that had been modified by CA-AuNPs. After 5 min, the change in the color of the paper was observed. Each paper was photographed and then analyzed for comparison of the intensity of the blue and red colors using ImageJ software.

### 3.8. Application of Colorimetric Paper Sensor in Water Samples

The lake water sample solution at the Universitas Indonesia, Depok, was filtered through a 0.45 µm membrane. Water was taken as samples from Kenanga Lake, FMIPA Lake, and Mahoni Lake. The three lake waters were then spiked with 0 M and 0.01 M Hg^2+^ and dripped onto a paper set immobilized by CA-AuNPs. 

## 4. Conclusions

A colorimetric sensor made from filter paper immobilized with gold nanoparticles for the detection of Hg^2+^ in water samples has been developed. This colorimetric sensor device was made by functionalizing gold nanoparticles using cyanuric acid. A clear color change from pink to bluish-purple was shown by this CA-AuNPs paper sensor in water with a fairly small concentration of Hg^2+^, up to 0.05 µM. This colorimetric response was also selective towards Hg^2+^ after being tested with different metal ions. The observed color change occurred due to a reaction with cyanuric acid to form CA-Hg^2+^-CA and reduce the stability of CA-AuNPs. The paper sensor can be used as the fast, portable, and reliable sensor for the detection and monitoring of Hg^2+^ levels in water.

## Figures and Tables

**Table 1:** Effect of CA-AuNPs immobilized paper type on grayscale intensity and absorbance.

Paper Type	Hg^2+^ Concentration	Grayscale Intensity	Absorbance
Whatman No. 1	0 M	103.231	0.3927
	0.1 M	105.800	0.3821
Whatman No. 41	0 M	123.203	0.3159
	0.1 M	136.244	0.2722
Whatman No. 91	0 M	107.775	0.3740
	0.1 M	108.605	0.3707
Whatman Chromatography No. 1	0 M	122.709	0.3177
	0.1 M	119.341	0.3298

**Table 2:** Effect of CA-AuNPs immersion time on paper with grayscale intensity and absorbance.

Hours	Hg^2+^ Concentration	Grayscale Intensity	Absorbance
3 h	0 M	133.500	0.2811
	0.1 M	120.974	0.3238
6 h	0 M	132.767	0.2835
	0.1 M	121.400	0.3223
12 h	0 M	125.072	0.3094
	0.1 M	117.704	0.3357
24 h	0 M	103.231	0.3927
	0.1 M	105.800	0.3821

**Table 3:** Effect of drying temperature on intensity of various complementary colors.

Temperature	Hg^2+^ Concentration	Intensity				
		Red	Green	Blue	Blue/Red	Blue/Red (0.1 M/0 M)
30°	0 M	113.100	97.081	100.203	0.8860	1.0690
	0.1 M	111.343	100.402	105.454	0.9471	
50°	0 M	174.457	154.375	157.355	0.9020	1.0837
	0.1 M	157.472	145.819	153.921	0.9774	
75°	0 M	113.963	101.226	104.007	0.9126	1.0063
	0.1 M	133.145	115.635	122.273	0.9183	
100°	0 M	123.709	110.631	112.694	0.9110	1.0180
	0.1 M	126.754	113.248	117.549	0.9274	

**Table 4:** The blue/red intensity of CA-AuNPs-immobilized paper exposed to various metals at a concentration of 0.1 M by ImageJ.

Metal Ions	Intensity			
	Red	Green	Blue	Blue/Red
Hg^2+^	173.321	161.452	161.614	0.9325 *
Mg^2+^	167.572	147.820	148.230	0.8846
Fe^2+^	198.262	177.329	167.888	0.8468
Cd^2+^	194.926	167.481	173.015	0.8876
Zn^2+^	201.482	176.317	180.412	0.8954
Ag^2+^	190.155	160.861	170.877	0.8986
Ba^2+^	175.092	150.728	150.532	0.8597
Mn^2+^	210.09	182.610	183.489	0.8734

Note: * according to one-sample *t*-test, *p*-value < 0.001 means that the blue/red intensity from Hg^2+^ and other metal ions is significantly different.

**Table 5:** The results of the analysis on lake water samples.

No	Lake Water Sample	Hg^2+^ Addition	Intensity			
			Red	Green	Blue	Blue/Red
1.	Kenanga	0 M	147.418	127.108	128.371	0.8708
		0.01 M	169.476	157.839	161.568	0.9533
2.	FMIPA	0 M	143.432	128.982	128.546	0.8962
		0.01 M	180.410	164.549	168.933	0.9364
3.	Mahoni	0 M	155.028	136.602	136.415	0.8799
		0.01 M	184.737	168.058	174.036	0.9421

## Data Availability

Not applicable.
